# Integrating Community Service into Student Learning: A Model Event of a Plastic Waste Cleanup

**DOI:** 10.1021/acs.jchemed.4c01164

**Published:** 2025-01-23

**Authors:** Jin Qian, Mikaela Sadri, Sara Valdez, Claire Clemons, Zhe Qiang

**Affiliations:** # School of Polymer Science and Engineering, The University of Southern Mississippi, 118 College Drive, Hattiesburg, Mississippi 39406, United States

**Keywords:** General Public, Public Understanding, Outreach, Hands-On Learning, Manipulatives, Materials Science

## Abstract

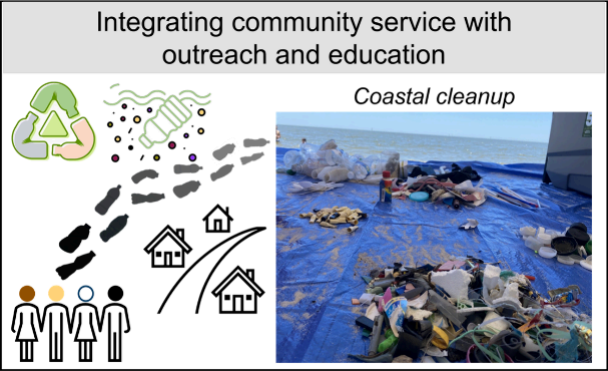

Plastic recycling has gained increasing attention due to the negative impacts of improper plastic waste management and its end-of-life outcomes. Despite growing research and educational efforts on sustainability, the integration of community service into student learning experiences remains limited. To address this gap and promote sustainable practices among the younger generation, a cleanup outreach event is developed in conjunction with pre- and post-cleanup lectures. The lectures covered the knowledge of plastic waste and recycling, relevant policies, and advancements in sustainability within industry and academia. The waste cleanup activity, held at Biloxi Beach and Hattiesburg, Mississippi, provided students with hands-on experience in addressing local plastic pollution and connected classroom learning to real-life plastic waste issues. Integrating community service with educational content provides an approach to learning about sustainable practices while raising awareness of societal needs and future technological opportunities.

## Introduction

Plastic materials have become ubiquitous across various industries due to their combined advantages of low cost and satisfactory material properties.^1,2^ However, their vast production and use have raised significant concerns regarding their environmental and human health impacts, particularly at the end of their life cycle.^3,4^ Unfortunately, a substantial portion of plastic waste is improperly disposed, which ultimately contaminates environmental ecosystems. Since most commodity plastics are resistant to complete natural degradation, they persist in the environment for extended periods of time (e.g., at least decades), while experiencing various aging processes and breaking down into microplastics.^5^ Both large plastic debris and microplastics can severely harm marine life by causing injury or death through entanglement, ingestion, and disruption of marine ecosystems.^6^ These microparticles have been detected in oceans, soils, the atmosphere, and within food chains,^7−10^ posing potential health threats to humans through inhalation and ingestion.^11^ The persistent nature of these plastics, combined with their tendency to fragment into microplastics, indicates the urgent need for more sustainable practices and solutions.^12^

In recent years, significant efforts have been directed toward K-12 education and outreach on the topic of plastic sustainability, with the goal of increasing student awareness of the challenges and opportunities in this area.^13−15^ For example, Lim et al. introduced laboratory experiments on polyethylene terephthalate (PET) depolymerization via aminolysis and the reuse of cleaved PET fragments into synthesizing new polymers in multiple high schools.^16^ These activities can enhance student knowledge and inspire them to pursue research aimed at addressing these societal challenges. However, it is also recognized that strong community engagement is crucial for fostering behavioral changes, particularly in how individuals dispose of plastics. A recent report by Milne et al. described how educating the public about microplastics through a museum exhibit increased visitor awareness of microplastic pollution, suggesting the importance of community engagement.^13^ Despite the growing focus on sustainability, integrating community service into student learning experiences, specifically on the topics of plastic waste management, remains underexplored. Developing these activities could play a pivotal role in promoting sustainable practices among younger generations, particularly undergraduate students. By encouraging a more informed and responsible approach to plastic use and disposal within communities, such initiatives could also help connect students to emerging research areas in sustainability.

Recognizing that our students often have limited firsthand experience with effective management of real-world plastic waste, an outreach event was organized to bridge this gap, which consisted of two lectures and a cleanup activity, each designed to connect classroom learning with practical, real-life issues surrounding plastic waste (Table 1). The pre-cleanup lecture provided foundational knowledge on plastic waste and microplastics, preparing students for the hands-on cleanup activity conducted in Biloxi or Hattiesburg, Mississippi, and also gave insight into their prior knowledge on the topic. Following the cleanup, a post-cleanup lecture highlighted recent advancements and opportunities within the plastic recycling industry, offering students awareness of potential solutions and innovations. The feedback from students provides valuable insights into the impact of our outreach event, in addition to informing future program design that integrates classroom education with practical experience in plastic waste management. This event also gave students the opportunity to reflect on the cleanup itself and the negative environmental impact of plastic waste on the environment.

**Table 1:** Outline of the Activities, Timeframes, and Key Takeaways for Each Day

Day	Activities	Time	Key takeaways
Section 1 – Before Cleanup	• Pre-activity questionnaire	45 min	• Foundational knowledge of plastic waste and recycling
	• Pre-cleanup lecture		• Formation and cumulation of microplastics
			• Common misconceptions and clarifications about recycling
			• Cleanup basics: what to expect and what to bring
Section 2 – Cleanup	• Cleanup activity	1.5 h	• Hands-on experience with collecting and sorting plastic waste
	• Sorting of collected waste		• Understanding the waste composition of a populated local coastal or suburban region
Section 3 – After Cleanup	• Post-cleanup lecture	45 min	• Summary of the cleanup event
	• Post-activity questionnaire		• Local to global cleanup events
			• Recycling policies
			• New technologies and industrial efforts
			• Academic recycling efforts

## Strategy and Designing

There were two cleanup events including a Coastal and Community Cleanup. The Coastal Cleanup took place at The University of Southern Mississippi (USM) and Biloxi Beach and involved 27 participants (9 undergraduate students, 11 graduate students, and 7 faculty/staff) over a 3-day period. For this cleanup, the graduate students and faculty/staff were volunteers from the School of Polymer Science and Engineering at USM and the undergraduates were affiliated with the department through a summer program. The first day consisted of an ∼45 min (pre-cleanup) lecture that covered the origins of micro- and ocean plastics, what types of plastics can be recycled as well as current challenges within recycling, and what to expect on the day of the cleanup (e.g., the roles of the collector/recorder described in the Coastal Cleanup section). The second day involved the Coastal Cleanup/sorting of the trash collected. The third day was comprised of an ∼45 min (post-cleanup) lecture covering how individuals could help tackle the plastic waste problem including other local cleanups/how to find them, recycling programs available for a variety of item types (e.g., clothing, electronics, etc.), and advancements in sustainability within industry and academia. The Community Cleanup took place at USM and Longleaf Trace in Hattiesburg, MS. For the Community Cleanup, the participants included 25 undergraduate students in a USM materials course for non-science majors over a 2-day period. The first day consisted of the pre-activity questionnaire, pre-cleanup lecture, and cleanup. Additionally, the first day was split up into two groups (each consisting of ∼1/2 the class) during their lab periods (2 h and 45 min). The second day consisted of the post-cleanup lecture and post-activity questionnaire and was done once for the class in its entirety during a lecture period (50 min). While the lectures were tailored to the knowledge level of the two groups, the learning objectives for these events remained the same and include:

1. Increase knowledge of microplastics,
2. Learn effective methods for recycling household plastics,
3. Gain insight into challenges of recycling, and
4. Recognize individual actions that can help reduce/manage plastic waste.

The goal of this project also includes inspiring participant interest in opportunities focused on addressing plastic waste challenges for a more sustainable future.

## The Cleanup

### Coastal Cleanup

The Coastal Cleanup occurred at Biloxi Beach, MS, a public beach in Harrison County on June 22, 2024. There was a total of 27 volunteers cleaning for 1.5 h. Within that time, approximately three large trash bags (capacity: 30 gal) were filled with waste across 0.75 mi of the coastline. Upon arrival to Biloxi Beach, volunteers met at the sign-in station where they were able to find a partner and get their supplies. At this station, the data collection sheet (provided in Supporting Information), two-bucket system, and safety procedures were explained to ensure a safe and effective coastal cleanup. Specifically, volunteers were paired together, where one person was the recorder, and the other was the collector. Each pair was equipped with the following items: a data collection sheet, clipboard, pencil, one grabber, two collection buckets, and gloves. As a main area of focus was understanding the composition of the collected trash from the beach, one bucket was designated for plastic and film waste, while the other bucket was for all other types of waste, which eliminated one of the sorting steps after collection and ultimately increased efficiency. The recorder was assigned to record all collected items into the appropriate category within the data collection sheet, and the collector was charged with picking up each trash item and performing an initial sort between the “plastic/film” and “other” buckets. The official beach cleanup and collection lasted for 1–1.5 h. At the end of the designated time, each pair returned to the sign-in area, where their bucket contents were weighed and set aside for sorting. It should be noted that in addition to the data forms, clipboards, pencils, grabbers, buckets, and gloves, the sign-in station also had a first-aid kit, hand sanitizer, large plastic trash bags, a scale, a folding table, tarps for sorting trash and a pop-up canopy tent (Table 2). Each of these items were provided by the Mississippi State Univerisity (MSU) Coastal Research and Extension Center, excluding the table, tarps, and canopy. Additionally, volunteers were encouraged to bring sun protection including sunscreen, a hat, and sunglasses, in addition to a reusable water bottle.

**Table 2:** List of Supplies Important for a Successful Cleanup

At sign-in station	With each collection pair	For individuals to provide
First-aid kit^a^	Data collection forms^a^	Sunscreen
Hand sanitizer^a^	Clipboard^a^	Hat
Large plastic trash bags^a^	Pencil^a^	Sunglasses
Scale/balance^a^	Grabber^a^	Reusable water bottle
Folding table (opt.)	Buckets^a^	
Tarpaulin (opt.)	Gloves^a^	
Pop-up canopy tent (opt.)		

a Items provided by the MSU Coastal Research and Extension Center for the Coastal Cleanup.

### Coastal Sorting Results

**Figure 1:**
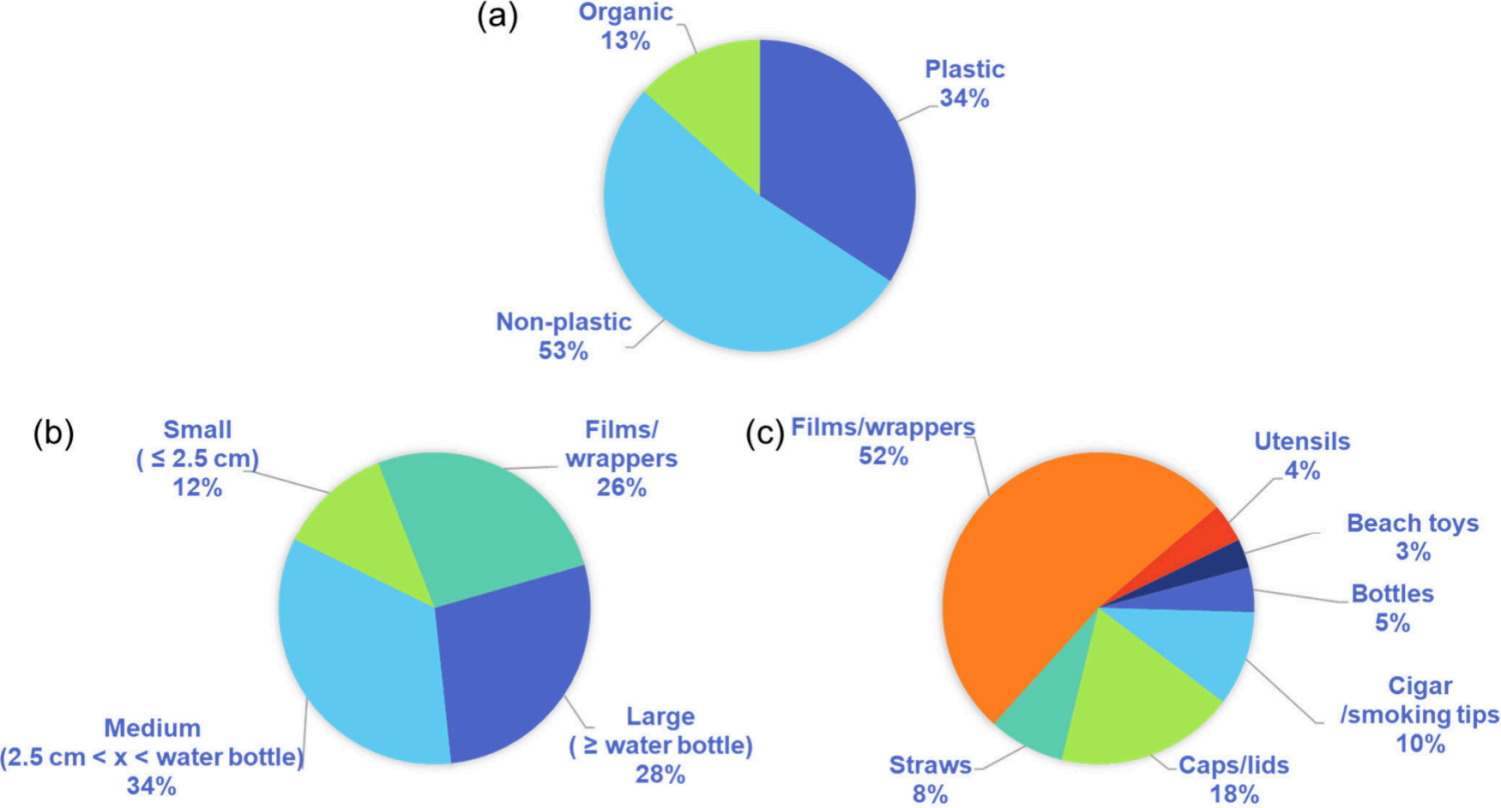
(a) Composition of all litter picked up from the beach including organic waste (13 wt %), plastic waste (34 wt %), and non-plastic waste (53 wt %). (b) Size composition of total plastic waste a function of mass (small, medium, large, and wrapper/films), and (c) composition of identifiable plastic waste by count of individual items.

**Figure 2:**
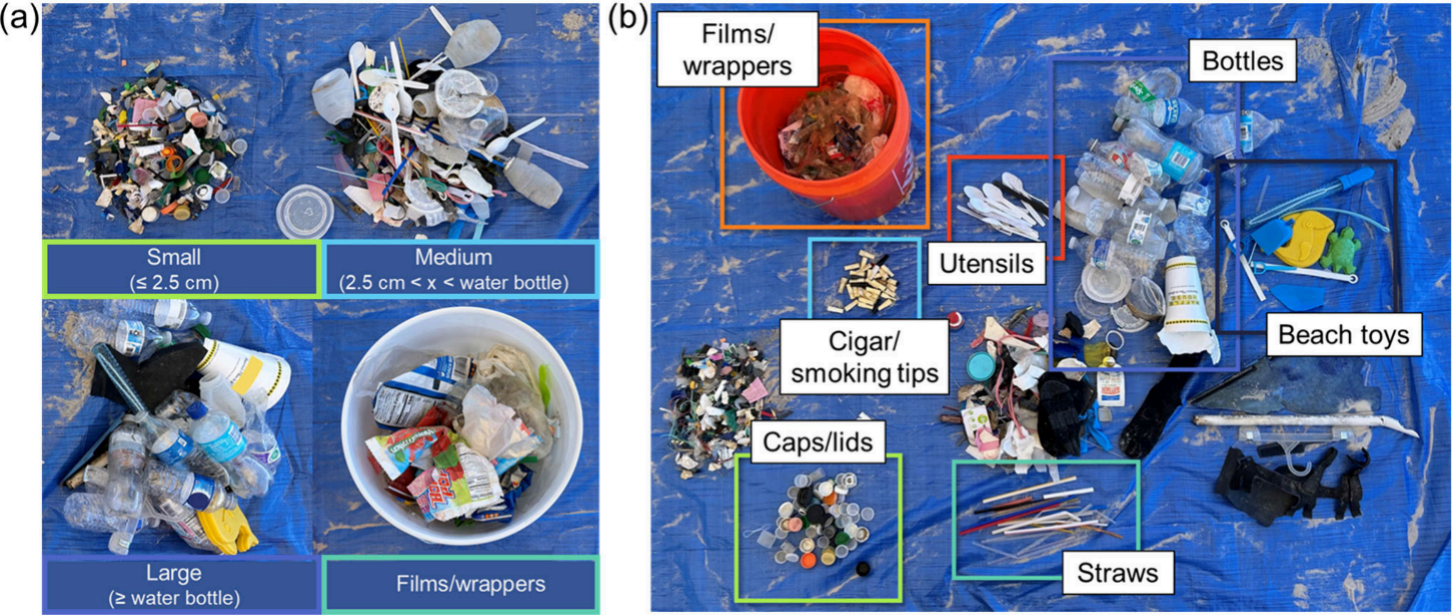
Specific waste piles for the different sorting, where (a) corresponds to separation by size, and (b) categorized identifiable objects together by type and count.

All collected trash from the cleanup activity was brought back to the sign-in station and each bucket with presorted contents was weighed and categorized into: 1) plastic/film waste, 2) non-plastic waste, and 3) organic waste. As shown in Figure 1a, 34 wt % of the waste was from plastic products, 53 wt % was other non-plastic trash, and 13 wt % was organic waste including a fish and some potatoes. At this point, the non-plastic and organic wastes were introduced into large trash bags and disposed of properly, and the plastic waste was kept to sort in two additional ways to better understand the composition. To start, all plastic waste was separated into four distinct categories, as shown in Figure 2a, including 1) small items, smaller than 2.5 cm, 2) medium items, larger than 2.5 cm but smaller than a water bottle, 3) large items, larger than a water bottle, and 4) all wrappers and films. While the wrappers and films collected had various sizes ranging from small to large, we found that sorting them at the cleanup site was difficult due to their lightweight (and how windy it was on the coast). As a result, for each sorting, we categorized them all together to prevent losing them back into the environment. Analyzing the sizes of the collected plastic waste, it was found that small items constituted the smallest percentage by mass at 12 wt % (Figure 1b). Wrappers accounted for 26 wt % of the plastic waste, followed by large items at 28 wt %. However, the majority of the plastic waste consisted of medium-sized items, making up 34 wt % of the total plastic collected along ∼0.75 mile of coastline within 1 h. Understanding the size scale of the trash is important when students are interested in how to prevent and respond to microplastic formation from occurring in coastal areas. The identifiable collected plastic waste was also sorted by general category to understand what most commonly was left on or washed up onto Biloxi Beach within the cleanup area, where the collected items are shown in Figure 2b. These categories, in order of most common to least common by count (Figure 1c), were: 1) wrappers/films (191, 52%), 2) caps and lids (68, 18%), 3) cigar/smoking tips (36, 10%), 4) plastic straws (29, 8%), 5) bottles (17, 5%), 6) eating utensils (15, 4%), and 7) beach toys (11, 3%). Overall, based on mass and count, the majority of the plastic waste found on Biloxi Beach appeared to be related to food and beverage consumption, which were potentially discarded improperly.

### Community Cleanup and Sorting Results

The Community Cleanup took place on the Longleaf Trace biking trail in Hattiesburg, MS beginning at the Longleaf Trail Gateway Parking on November 15th and 19th, 2024. There were a total of 25 participants across the two lab sections, cleaning for a total of 1.5 h. Within that time, five lawn and leaf trash bags (capacity: 39 gal) were filled with waste across 0.3 mi, with a total mass of 41.3 kg. As the Community Cleanup occurred directly following Section 1 (pre-activity questionnaire and pre-cleanup lecture), the participants walked over to the Longleaf Trail Gateway Parking lot together to meet an organizer who drove to the location and organized supplies for the appropriate number of pairs from the trunk. All participants were informed of the safety procedures and methodology for a safe and effective cleanup as described within the Coastal Cleanup section back at the classroom. As such, they were able to quickly pair up, assign roles, gather equipment, and begin collecting immediately upon arriving to the location. It should be noted that the two-bucket system was not utilized within these groups due to the limited number of buckets since the Community Cleanup did not use equipment provided by MSU Coastal Research and Extension Center due to the distance from the coast. The official collection time for each lab section was 45 min. Participants met back up at the car at the end of the cleanup time or anytime their bucket was full in order to get a weigh-in and empty their buckets for additional collection, where a supervisor was stationed in order to facilitate.

**Figure 3:**
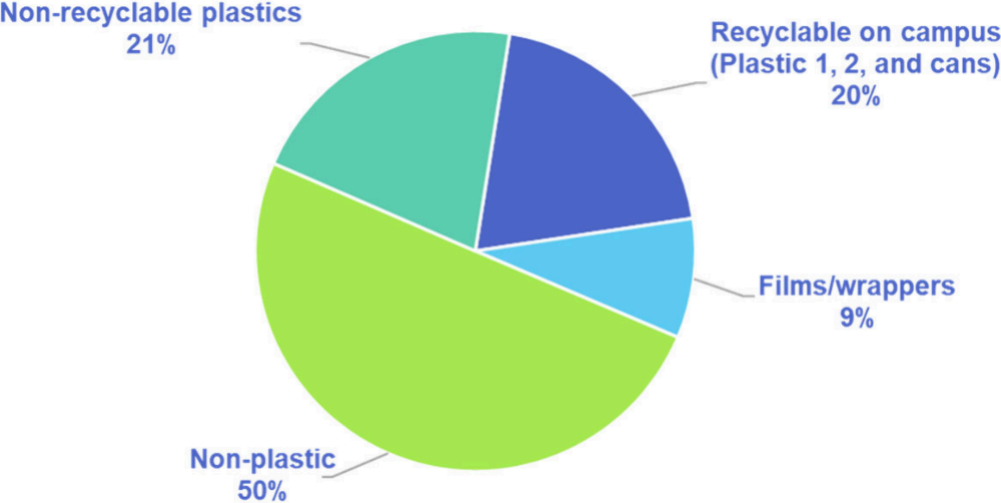
Composition of litter picked up from the Community Cleanup in the first lab section by weight, including non-recyclable plastics (21 wt %), recyclable on campus which are cans as well as plastic 1 and 2 items (20 wt %), films/wrappers (9 wt %), and other non-plastic items (50 wt %).

At the end of the Community Cleanup activities, all of the contents were weighed and then sorted. The collected litter was sorted into four different categories: 1) non-recyclable plastic, 2) items and materials recyclable on campus (plastic 1, 2, and cans), 3) films/wrappers, and 4) non-plastics. As shown in Figure 3, it was found that 50 wt % of the litter were non-plastics, 21 wt % were non-recyclable plastics, 9 wt % were films/wrappers, and 20 wt % was made up of materials which are recyclable on campus. After sorting and weighing the litter, it was then separated into “recyclable” and “non-recyclable.” The non-recyclable items were placed into a nearby dumpster while the recyclable items were placed into a campus recycling bin on the way back to the classroom. Through this exercise, the students were able to learn about the general composition of waste materials around them and understand how much of what is normally thrown out improperly can be recycled within their own community, all while participating in community service.

### Improvements

Overall, both the Coastal and Community Cleanups were successful and removed nearly 50 kg of all types of trash across 0.75 mi of Biloxi Beach coastline and 0.3 mi of the Longleaf Trace in 1.5 h at each site. However, there were some notable areas of improvement that were made upon conclusion of the Coastal Cleanup and the beginning of the Community Cleanup, including:

- more research into choosing a cleanup location,
- more discussion and better explanation of what common items are made of plastic (e.g., clothing fibers or multilayer food packaging), and
- recycling potentially recyclable items, when possible.

The cleanup area chosen for the coastal event was on a heavily populated beach maintained by the city of Biloxi with trashcans approximately 100–200 ft apart for the entire length of the coastline we collected over. Thus, the majority of the trash collected was likely from trashcan overflow or littering. However, there is a better opportunity to make a difference if the area collected over were in a location with fewer trashcans and/or collected wash-up from the water. This was addressed when transitioning to the Community Cleanup on Longleaf Trace, beginning at the Longleaf Trail Gateway Parking lot. Despite being a well-populated area located across from the university and directly behind dormitories, there were very few trashcans within the cleanup area (1–2 across the entire area). Furthermore, the brush near and around the trail collected a significant amount of debris from the rain wash-up.

During the initial Coastal Cleanup, volunteers were sent out to collect trash in pairs, separating the plastic and non-plastic items as they went. However, upon returning back to the main camp for weigh-ins and sorting, it appeared that many groups did not understand whether an item was plastic or not. As a result, some additional resorting had to be performed. This was addressed in the Community Cleanup by 1) including some examples within the pre-activity slides when explaining what to expect, and 2) by eliminating the two-bucket system. The initial reason for eliminating the two-bucket system was due to a limited supply of buckets, however, by encouraging the students to put everything into a single bucket, it provided a chance for more discussion when sorting all waste at the end. At that point, real-world examples of common litter and its material could be used to demonstrate the commonality of plastics within our world.

Finally, upon the conclusion of the sorting process within the Coastal Cleanup, all of the collected waste was placed into large trash bags and then disposed of into a nearby dumpster. Due to the beach location, there were no accessible recycling bins to further increase our positive impact on the area. However, due to the proximity of the Community Cleanup to the USM campus, all of the recyclable items and materials were sorted from the rest of the trash and disposed of into the appropriate recycling bin. The recyclable materials made up 20 wt % of the collected waste, as discussed previously. We believe that this change allowed the students to better recognize recyclable items in the future and may have attributed to the success of the second iteration of the activity, which will be further explained in the Community Cleanup Test Lesson.

## Lecture Results

### Pre-cleanup Lecture

The pre-cleanup lecture was designed to provide students with knowledge about plastic waste, recycling, and the upcoming cleanup activity. The session began by discussing the current state of plastic production and disposal, highlighting that a large portion of plastic waste ends up landfilled (around 45%), incinerated (about 17%), or randomly discarded (around 22%),^17^ leading to environmental damage through carbon emissions and microplastic formation. For non-science major students, additional background knowledge about polymers was provided to help them better understand the subsequent content. Following this, the concept of microplastics, including their sources, formation processes, and how they circulate through the food chain was introduced, emphasizing the necessity of recycling to reduce the reliance on incineration and landfills. While developing biodegradable plastics offers a potential route to sustainability, plastic recycling remains the most viable solution for addressing the vast amounts of existing plastic waste.^18−20^ Therefore, two primary recycling methods, mechanical and chemical recycling, were introduced. In brief, mechanical recycling is the most used method currently, which involves reprocessing waste materials into new products while maintaining their chemical identity. However, this method requires extensive sorting and often results in downgraded mechanical properties of the recycled plastics during the reprocessing.^21,22^ The typical process includes pre-sorting, coarse shredding, washing and drying, fine shredding, sink-float sorting, and extrusion into new products. In contrast, chemical recycling offers the advantage of requiring less sorting and potentially yielding higher-value products, though its high energy cost limits its application in the recycling industry.^23,24^ For more informed students, we recommend expanding this topic further with more specialized knowledge. An introduction to two recycling terminologies, *incineration* and *pyrolysis*, was included in the provided materials as an example. Next, common misconceptions about recycling were addressed and discussed (e.g., compostable vs recyclable, wish-cycling, plastic bottle caps being recyclable, etc.), ensuring that students had a clear and accurate understanding. Finally, the cleanup event was introduced, explaining its purpose and logistics to prepare students for the following activities. Overall, the pre-cleanup lecture aimed to help students understand recycling and proper waste management while encouraging active participation in the cleanup activity.

### Post-cleanup Lecture

We provided a post-cleanup lecture to conclude the cleanup event and enhance student understanding of individual actions to mitigate plastic waste. The lecture covered a summary of the cleanup event, related activities and policies addressing plastic waste, and advancements in plastic recycling within industry and academia. The goal for the post-cleanup session was to connect classroom learning to real-world applications and inspire participants to explore the opportunities in plastic waste management.

#### Part 1: Local and National Plastic Waste Control Projects

The first section of the lecture introduced plastic waste control projects, focusing on cleanup events and recycling programs. We started with the cleanup events in the local areas, for which dates and registration details were provided in the slides for students who were potentially interested in participating. Organizations hosting community cleanup events in the US and globally were also listed for students outside the local areas. Next, the lecture covered the recycling services available through the university and the city of Hattiesburg, MS. We provided the instructions for registering with these recycling services and listed the acceptable and unacceptable waste items. It was noted that some items, such as glass, clothing, and plastic bags, were not accepted by either the city or campus recycling services. Therefore, local stores that accept a broader range of plastics and companies offering mail-in recycling services were introduced. Overall, this section aimed to inform students about local events and projects related to plastic waste management and what individuals can do to reduce the adverse impact of plastic after their end-of-life.^25−27^ By sharing this information, the lecture sought to inspire active participation and civic engagement in environmental preservation efforts, empowering participants to make meaningful contributions to their communities and beyond.

#### Part 2: Policy for Recycling

The second section focused on government-level efforts to manage plastic waste such as recycling policies. We introduced the policies from the local level of Mississippi state and other states to the federal and global efforts, aiming to provide students with a comprehensive understanding of the broader context. We also discussed the distinct recycling policies of states like California, New York, and Massachusetts, which include mandatory recycling and extended producer responsibility (EPR) programs.^28,29^ At the federal level, the Sustainable Materials Management (SMM) and National Recycling Strategy, promoted by the Environmental Protection Agency (EPA), were introduced, which aimed at enhancing recycling by creating a more sustainable recycling system. This information helps participants understand the future direction of recycling development. Finally, representative recycling policies that occur on the global level, such as mandatory waste sorting regulations, EPR, and pay-as-you-throw systems, were discussed to highlight the strong government efforts to address the plastic waste challenge.

#### Part 3: New Technologies and Industrial Efforts in Recycling

The third section discussed recent developments in plastic waste recycling technologies and industrial actions aligning with EPA policies to tackle plastic waste. We introduced three emerging recycling technologies and approaches, providing one or two examples for each. The first was a chemical recycling process that converts the waste plastic into its original building blocks or new products by breaking chemical bonds through high-temperature treatment.^30^ The second technology was a high-speed robotic sorting system guided by artificial intelligence (AI), which allows faster and more accurate sorting than the manual methods, thus improving the efficiency and profitability of the mechanical recycling industry. The third innovation was a third-party platform that partnered with a variety of well-known brands to design and produce refillable containers for products that are usually sold in single-use packaging. This platform further managed the collection, cleaning, sanitizing, and redistribution of these containers from the customers back to producers, fostering a circular ecosystem between producers and users.

It is important to note the growing social consciousness in the industry, with more companies integrating sustainability-focused practices into their supply chain, product design, and marketing.^31^ For example, in the food and beverage industry, leading enterprises are setting goals to reduce plastic waste by making their packaging recyclable and using recycled materials.^32^ In packaging design, there is a growing trend of using single-type plastics instead of multilayer, multicomponent films to enhance recyclability.^33^ In the clothing industry, sustainable brands are increasingly offering recycling services and designing products from recycled plastics.^34^ Overall, this section aimed to showcase the industry’s latest advancements in addressing plastic waste.

#### Part 4: Academic Efforts in Recycling

**Figure 4:**
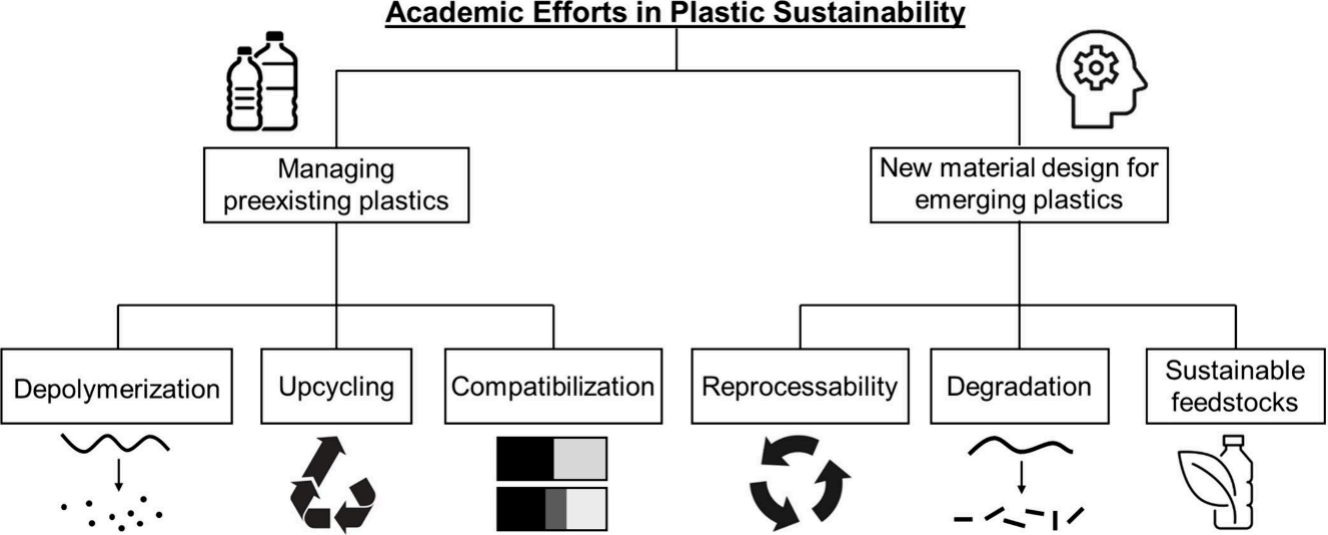
Outline of academic efforts in plastic sustainability.

The goal of part four was to motivate students to pursue academic and research opportunities that contribute to sustainability by highlighting cutting-edge academic efforts on recycling technologies. Figure 4 outlines this section, which covers academic progress in recycling preexisting plastics and designing new materials for emerging plastics. Mechanical and chemical recycling are the primary strategies for addressing preexisting plastics. Over the past two decades, significant efforts have been made to develop new recycling technologies to address existing shortcomings.^35−38^ One key approach involves the compatibilization of plastic blends, which stabilizes the interface between two immiscible plastics and improves their miscibility.^36^ This approach is critical for mechanical recycling, as immiscible plastics often produce materials with poor physical properties. Well-designed compatibilizers can significantly enhance recycling efficiency by producing high-performance material from mixed plastic waste and reducing the need for labor-intensive sorting in the traditional mechanical recycling process. The second academic example focused on depolymerization, a chemical recycling process that breaks down plastic waste into its basic building blocks (monomers or oligomers). Common methods for depolymerizing commodity polymers include pyrolysis and catalytic chemical depolymerization, while hydrolysis is mainly used for polyesters like PET.^39^ Depolymerization of plastic waste offers a sustainable solution for managing plastic waste by enabling the recovery of reusable raw materials, which can be reintegrated into production cycles. The third example focused on plastic waste upcycling, which involves various chemical and mechanical methods of converting waste plastic into value-added products. Academic research has developed numerous upcycling techniques that hold the potential for fabricating new materials with desired properties from waste plastics.^40^

In addition to recycling efforts, this section explored the design of new materials that align with the principles of a circular economy. Advances in polymer research are increasingly focused on balancing functionality, recyclability, and environmental sustainability. These goals can be achieved by designing new materials from sustainable feedstocks and incorporating desired reprocessability and degradability.^41^ This section aimed to introduce students to the concepts of these strategies, while specific research examples were provided to undergraduates affiliated with the department to deepen their understanding, as they possessed more advanced backgrounds in polymers.^42−44^ Overall, this section introduced the cutting-edge academic progress in plastic sustainability with the intention of engaging students in research that addresses real-world waste challenges.

#### Part 5: Supplementary Content Requested by Students

This section provided additional content requested by students during the pre-cleanup lecture. Beyond the topics already covered in our scope, our students expressed interest in the impact of ocean plastic pollution. In response, we discussed how ocean plastic affects marine animals, ecosystems, and human health. As part of this discussion, the Nurdle Patrol program was introduced during the post-cleanup lecture.^45,46^ Briefly, nurdles are pre-production plastic pellets used as raw material in manufacturing plastic products. They often spill into the ocean during transportation, causing plastic pollution and harming aquatic life and the environment. Nurdle Patrol is a citizen science project led by the Mission-Aransas National Estuarine Research Reserve, which aims to collect data on nurdle distribution, remove them from the environment, and raise awareness about this issue. Here, we suggest activity designers tailor the content of this part to align with the students’ backgrounds and interests, ensuring that the lecture remains relevant and engaging.

The main lecture materials can be found in the Supporting Information and to make it easier to use, slide usage suggestions are provided in the Supporting Information and at the end of the slide deck.

### Coastal Cleanup Test Lesson

To assess the level of understanding and determine if the learning objectives were met, a post-activity questionnaire was given to the participants using an anonymous online form after the completion of the second, post-cleanup lecture. This outreach event was performed over a 3-day period with the Coastal Cleanup on day 2 and lectures on days 1 and 3, and of the 27 volunteers that attended the cleanup, only 11 filled out the questionnaire. Of the 11, there was one faculty/staff, 5 graduate students, and 5 undergraduate students. The authors believe that the low participation for the questionnaire is partially due to the questionnaire not being given during the lecture, instead the link was sent out after the post-cleanup lecture. The questionnaire in its entirety and the results can be found in the Supporting Information. However, due to there not being a questionnaire given before the activities (lectures and cleanup), no definitive conclusions can be made using the information gathered from the questionnaire as there is nothing to compare the respondents’ answers to (i.e., we do not know their base knowledge). Although, when asked, 91% of the respondents self-proclaimed to have learned “some” or “a lot” about microplastics and recycling (asked as two separate questions) after the lectures. Additionally, when asked, “How likely are you to take action to help decrease your impact on microplastics entering our environment/help remove ocean plastics?”, 91% answered “somewhat likely” or “very likely” leading the authors to believe that despite not being able to prove the learning objectives where met, the goal of inspiring participants to become interested in opportunities focused on addressing plastic waste challenges was achieved. This was further demonstrated by participant responses when asked “What can you do as an individual to help with the plastics problem?” with answers such as “recycle properly and encourage others to recycle properly,” “participate in clean-ups,” and “be cautious of what plastics I use and try to reduce how much I use.”

### Community Cleanup Test Lesson

After taking into account what went well with the Coastal Cleanup lectures/questionnaire and making improvements upon what did not go as well, another iteration of the activity (lectures and cleanup) was conducted with 25 undergraduate students at USM enrolled in a materials class for non-science majors. The Community Cleanup was done over a 2-day period where day 1 consisted of the pre-activity questionnaire, pre-cleanup lecture, and cleanup (done twice with two groups made up of approximately half of the class) and day 2 consisted of the post-cleanup lecture and post-activity questionnaire (done once with the entire class). Of the 25 participants, 13 attended all components of the events, 6 only attended day 1, and 6 missed portions or all of day 1. Furthermore, 19 students completed the pre-activity questionnaire and 19 completed the post-activity. However, they were not the same 19 students, so when analyzing the responses, to best determine if the learning objectives were met, only the responses from the students who attended all components of the activity are included when referring to the post-activity questionnaire results (all of the results for the Community Cleanup questionnaires can be found in the Supporting Information).

**Figure 5:**
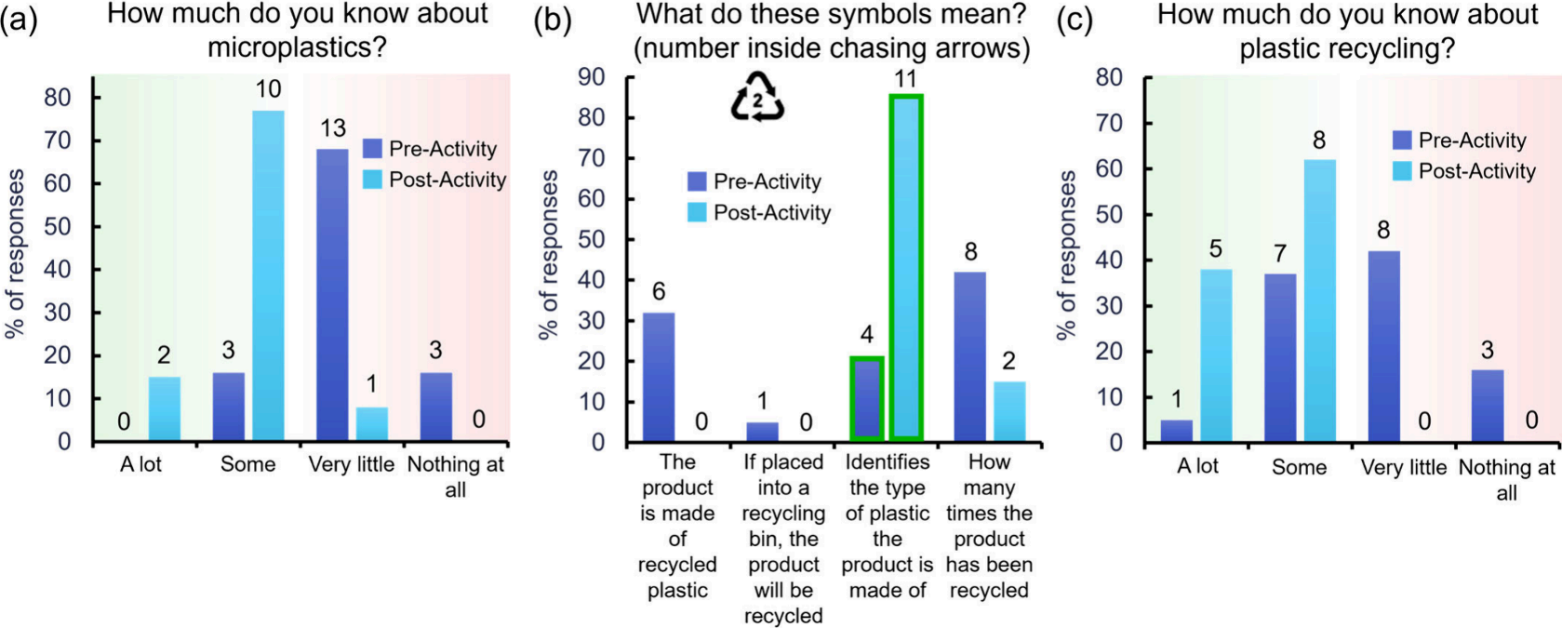
Results for the following questions from the questionnaires: (a) How much do you know about microplastics? (b) What do these symbols mean?, and (c) How much do you know about plastic recycling? The dark blue corresponds to the pre-activity questionnaire and the light blue to the post-activity. The numbers above each column are the number of responses to each answer choice.

To increase participation with the questionnaires, they were given on paper during the class meeting times. Several questions were asked to test if the first two learning objectives regarding microplastics and household recycling were met. These included: “How much do you know about microplastics?”, “What do these symbols mean? (number inside chasing arrows),” and “How much do you know about plastic recycling?” The results for these questions can be found in Figure 5.

In Figure 5a, we see that when asked about their understanding of microplastics, the majority of students answered with “very little” and “nothing at all” but after the lectures, over 90% claimed to know “a lot” or “some.” These results demonstrate that the lectures were effective at giving the students more knowledge of microplastics, thus the first learning objective was met (increase knowledge of microplastics). The question in Figure 5b probed if the students know what plastic/resin identification codes (RICs) are. Before the lectures, the answers varied between the four answer choices but after the activities, about 85% of the students were able to show that they know RICs identify the types of plastics (the correct answer, indicated by the green boxes in Figure 5b). The questionnaire also inquired about how much the students know about recycling, Figure 5c. After the activities a large shift can be seen with 100% of the students responding that they know “some” or “a lot” about recycling compared to the 42% that responded the same way before the activity. The results of the latter questions show that the second learning objective was also achieved (learn effective methods for recycling household plastics) as the knowledge of recycling in general was increased throughout the activity and knowing how to identify different plastics based off their RICs is a skill that makes household recycling more effective on both personal and industrial levels.

Learning objective 3 was to “gain insight into challenges of recycling” and this was addressed in the lectures by introducing and clarifying common misconceptions regarding recycling as well as issues related to society (e.g., recycling policies/laws in various countries) or the science related to the mixing of different plastics. To test if this learning objective was met, two questions were asked on the questionnaire, the first being, “If a plastic is advertised as being compostable, should you put it in the recycling bin?” Before the activities, 26% said “yes”, 21% “no”, and 53% said “I don’t know.” After the activities, 38% said “yes”, 47% said “no”, and 15% said “I don’t know.” While more students got the answer correct post lectures, more students also got it wrong without there being any definitive trend of learning. As a result, the authors would recommend placing a bigger focus on that misconception during the pre-cleanup lecture as the concept was not well understood. Another factor that could have influenced the results of this particular question is in the southern region of the United States, where USM is located, composting at a city- or state-level is not done, and the students were likely not that familiar with the concept of composting in general. The second question was open-ended and asked, “What do you think the challenges of recycling are?” Prior to the activity, some of the responses included, “not knowing how to categorize items”, “there’s a lot of rules and they vary based on where you live and recycling bins are not always accessible”, and “ensuring that it [waste] is properly recycled and not just dumped into a landfill, getting people to take initiative, and teaching what is and isn’t recyclable.” Post-activity, some of the responses were, “availability of recycling certain plastics in certain areas and the efficiency of recycling methods,” “sorting through what is actually recyclable or not,” and “no one is teaching the younger generations.” Overall, the responses to this question did not change much after the lectures were given and the majority of the responses focused on challenges on the individual-level (e.g., people not caring or knowing how to recycle) and the authors found this interesting. As mentioned earlier, the lectures also discussed recycling challenges as a society and the specific science involved, but these were hardly mentioned at all in the students’ responses. Additionally, the vast majority of the plastic waste problem itself is not caused at the individual-scale, yet the students still placed the “blame” on individuals. Collectively, we believe the third learning objective was only met with minimal success due to the persistent confusion with common recycling misconceptions, however, with the knowledge gained throughout this project, we also believe that with the suggestions made, in the future this could be done more effectively.

The final learning objective was to recognize individual actions that can help reduce/manage plastic waste. To assess the success of this objective the following question was posed: “What can you do as an individual to help with the plastic waste problem?” Answers before the activity included, “learn more about it so I can teach others and also be mindful of my waste”, “use less plastic, repurpose plastic, and recycle plastic after use”, and “an individual can minimize plastic usage on all fronts and make sure to properly recycle when plastic usage is absolutely necessary.” Post-activity, some of the responses were, “clean up any trash I see on the ground”, “I can understand what can and can’t be recycled and can encourage others to recycle”, and “do my part and recycle properly.” Similarly, we do not see much of a difference in the complexity/specificity of the responses, but in this case, we believe it is because most people probably know that to help with the plastic waste problem they should recycle and pick up trash. Therefore, we cannot say that we “achieved” this specific learning objective as the students already had this knowledge. However, with the lectures given, the students received more information, resources, and support so they can better execute these activities (e.g., how to look and sign up for future cleanups and how to determine what can be recycled in their area).

**Figure 6:**
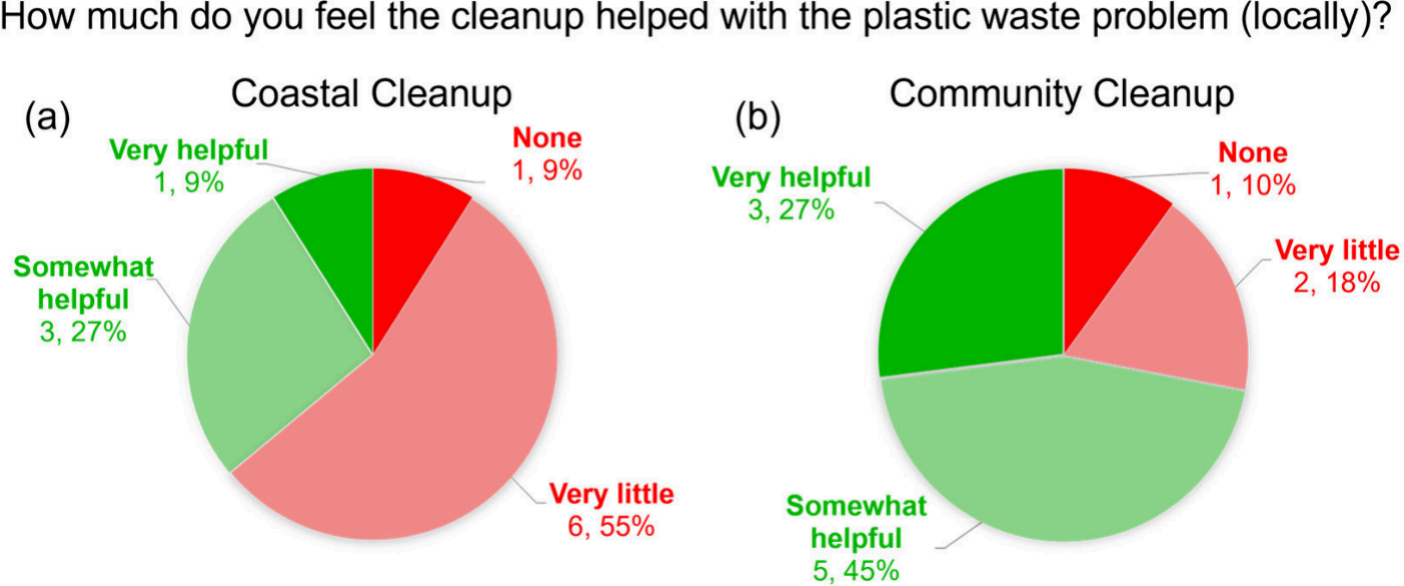
Charts showing the responses for the question “How much do you feel the cleanup helped with the plastic waste problem (locally)?” for (a) the Coastal Cleanup and (b) the Community Cleanup highlighting the number of responses for each choice and the resulting percentage.

Another interesting reaction we found is for a question posed in the post-activity questionnaire for both the Coastal and Community Cleanups which asked, “How much do you feel the cleanup helped with the plastic waste problem (locally)?” The results can be found below in Figure 6.

We see that the participants from the Community Cleanup responded much more positively to this question than those that participated in the Coastal Cleanup (72% vs 36%, respectively). We believe this was influenced by several factors including the authors taking action to implement the suggested improvements from the previous iteration of the cleanup as well as from the nature of the areas we were actually cleaning. In general, the area of the beach we cleaned was well maintained with trash cans available every couple hundred feet. Whereas the area of Longleaf Trace we cleaned had no visible trashcans, this location was chosen due to the combination of lack of trash cans and high foot traffic. Additionally, the waste found at the beach has been subjected to a harsher environment and weathering resulting in smaller pieces of trash that were partially concealed by the presence of sand. The trash collected from the Trace was in a less harsh environment and generally larger with nothing available to conceal it, so visually, the cleanup on the Trace looked like a more productive cleanup. In another attempt to incorporate the improvements, when we sorted trash at Longleaf Trace, we had a pile for plastics 1 and 2 and cans, items that are recyclable on USM’s campus, and upon completion of the cleanup when walking back to campus, we placed these items into the recycling bins. Overall, these results and efforts show that the improvements implemented to the cleanup were also felt and expressed by the participants.

## Conclusion

This work presents a model event toward an effort of integrating community service with outreach and education to enhance the young generation’s understanding of current recycling efforts and plastic waste management. The event included a cleanup activity and two lectures. The lectures focused on relevant policies for recycling, efforts in industry and academia, and individual actions to mitigate the plastic waste problem. The cleanup activities, held at Biloxi Beach and in Hattiesburg, Mississippi, provided the students with hands-on experience in addressing local plastic pollution and connecting classroom learning to real-life plastic waste issues. The event was implemented twice, allowing for iterative improvements. Pre- and post-questionnaires, along with quizzes, were used to assess student understanding of plastic waste and program outcomes. The main lecture slides, relevant documentation, and questionnaires with responses are available in the Supporting Information.

## Supporting Information Available

Coastal Cleanup Questionnaire, Community Cleanup Questionnaire, and Cleanup Lecture Slides (PDF)
