# Supplementary material for: Integrating Community Service into Student Learning: A Model Event of a Plastic Waste Cleanup
Source: J Chem Educ. 2025 Jan 23;102(2):661–70. doi: 10.1021/acs.jchemed.4c01164 (PMC11823409; doi:10.1021/acs.jchemed.4c01164)
Supplement: Supplementary file 1 — ed4c01164_si_001.pdf [file ed4c01164_si_001.pdf]

Supporting Information for:

Integrating Community Service into Student Learning: A Model Event of a Plastic Waste Cleanup

Jin Qian[∇], Mikaela Sadri[∇], Sara Valdez [∇], Claire Clemons, and Zhe Qiang[*]

[1] School of Polymer Science and Engineering, The University of Southern Mississippi 118 College Drive, Hattiesburg, MS 39406

* Corresponding Author (Email: zhe.qiang@usm.edu)

[∇]Co-first authors

COASTAL CLEANUP QUESTIONNAIRE (Post-Activity)

Number of participants: 11

1. Did you attend the pre and post cleanup lectures?
   A. Pre cleanup (0, 0%)
   B. Post cleanup (1, 9%)
   C. Both (9, 82%)
   D. Neither (1, 9%)

2. How old are you?
   A. 18-25 (6, 55%)
   B. 26-35 (4, 36%)
   C. 36-45 (1, 9%)
   D. 46-55 (0, 0%)
   E. 56-65 (0, 0%)
   F. >65 (0, 0%)

3. What is your highest level of education?
   A. K – 12 (0, 0%)
   B. High school degree (0, 0%)
   C. Some college (4, 36%)
   D. Bachelor's degree (1, 9%)
   E. Some graduate school (5, 46%)
   F. Master's degree (1, 9%)
   G. Doctorate (0, 0%)

4. How much did you know about microplastics/ocean plastics before the first lecture?
   A. Nothing at all (0, 0%)
   B. Very little (1, 9%)
   C. Some (9, 82%)
   D. A lot (1, 9%)

5. How much have you learned about microplastics/ocean plastics after the lectures (if applicable)?
   A. Nothing at all (0, 0%)
   B. Very little (1, 9%)
   C. Some (9, 82%)
   D. A lot (1, 9%)

6. What best describes how you felt about microplastics/ocean plastics before the first lecture?
   A. Calm (0, 0%)
   B. Somewhat calm (1, 9%)
   C. Neutral (2, 18%)
   D. Somewhat worried (6, 55%)
   E. Worried (2, 18%)

7. What best describes how you feel about microplastics/ocean plastics after the lectures and cleanup?
    A. Calm (0, 0%)
    B. Somewhat calm (1, 9%)
    C. Neutral (2, 18%)
    D. Somewhat worried (4, 36%)
    E. Worried (4, 36%)

8. How much did you know about recycling before the first lecture?
    A. Nothing at all (0, 0%)
    B. Very little (1, 9%)
    C. Some (8, 73%)
    D. A lot (2, 18%)

9. How much have you learned about recycling after the lectures (if applicable)?
    A. Nothing at all (0, 0%)
    B. Very little (1, 9%)
    C. Some (7, 64%)
    D. A lot (3, 27%)

10. What best describes how you felt about recycling efforts before the first lecture?
    A. Satisfactory (0, 0%)
    B. Somewhat satisfactory (1, 9%)
    C. Neutral (1, 9%)
    D. Somewhat inadequate (5, 45%)
    E. Inadequate (4, 36%)

11. What best describes how you feel about recycling efforts after the lectures and cleanup?
    A. Satisfactory (0, 0%)
    B. Somewhat satisfactory (2, 18%)
    C. Neutral (3, 27%)
    D. Somewhat inadequate (3, 27%)
    E. Inadequate (3, 27%)

12. Where do microplastics originate and what do you think the hazards of microplastics/ocean plastics are? (short answer)

    "microplastics originate from mechanical and chemical decomposition overtime from being handled/traferred/discarded. Microplastics could be disrupting delicate ecosystems as well as causing unforeseen health issues in humans and other animals further down the road"

    "Many places, anywhere plastic materials interact with the environment, largely from plastic discarded into the environment, fibers from washing clothes and groundwater pollution from landfills. Microplastics can enter the food chain at a variety of levels and circulate, the effects of plastic accumulation in the body can be many but none are positive, for example increased risks of birth defects and cancer in both humans and animals. This is in addition to increased greenhouse gas emissions, major disruptions of ecosystems, and ocean acidification."

"polymeric items of higher physical size (for example PET leached from clothes into washer drainage or trash discarded on a Mississippi beach)"

"They originate from plant spillage or trash. The hazards are they present dangers for sea animals and spreads microplastic waste that could be recycled."

"Microplastics originate from the many plastics that we use every day and the large amount of plastics that become trash and very slowly degrade into the environment. Plastics are not meant to be a part of the food chain and inevitably negatively impact us and our environment by causing microplastics to be part of every system."

"They can come from a multitude of things. But, they occur whenever plastic is broken down in an environment. They pollute the oceans as well as have negative impacts on wildlife and their environments."

"Is the easier question "where don't microplastics originate"? In modern society plastic is everywhere which means there are many, many sources of microplastics from paints and coatings to OEM waste to straight-up litter. These have demonstrable negative impacts on marine and terrestrial environments as hormone disruptors and sources of toxicity in some cases."

"They originate from larger plastics. A hazard is the pollution it is causing to the environment and the damage it can do to marine life."

"The breakdown of larger plastics"

"Homo sapiens are the root cause of excess microplastics, which are ultimately changing ecosystems to the detriment of our wildlife."

13. What do you think the challenges of recycling are? (short answer)

"Getting everyone to put in effort"

"Many, ranging from logistical to social. Cleaning, separation, collection, education, infrastructural support and effective legislative policy all present challenges for recycling efforts."

"in part economic and policy related. material property balance with recyclability"

"Too much inconvenience and money and time to recycle."

"People knowing what is okay to recycle and people recycling things like pizza boxes that make it difficult for the workers."

"Making people care about as well as there are few initiatives that are close by. Most people have to go out of their way to recycle. Also, recycling wasn't really focused on when we were kids."

"Some of the ongoing significant technical challenges are colorants, sortation, compatibilization; but there are also critical social, political, and economic factors that

also need to be addressed including simplifying recycling for consumers, incentivizing use of recyclate in manufacture, and EPR."

"Cost, space, society views"

"Sorting between different types of plastics"

"Proper sorting, contamination, & reprocessing"

14. What plastic items did you pick up at the cleanup? (e.g. straws, bottles, beach toys, etc.—short answer)

"lots of wrappers and plastic straws"

"Straws, small pieces of plastic, bottle caps"

"many types that were listed on the form, and many types that were not listed on the form"

"N/A"

"Straws, bottles, mostly small plastic pieces"

"I picked up straws, cups, candy wrappers."

"Food packaging was the most abundant recognizable item; but there were many many pieces of plastic that were simply too small to identify the source."

"cigarette butts"

"straws, bottles, bottle caps, plastics kid's toys"

"Cigarette butts, metal beer caps, plastic fragments, and food packaging."

15. What items of trash were most prevalent? (short answer)

"cigarette butts"

"Cigarette butts, bottle caps, straws"

"cigarettes as per the survey"

"N/A"

"Cigarette Butts"

"cigarette butts and random pieces of plastic"

"Cigarette butts, by far."

"Cigarette buts"

“Cigarette ends, dead animals, food”

“Cigarette butts and food packaging”

16. How much do you feel the beach cleanup helped with the plastics problem (locally)?
    A. None (1, 9%)
    B. Very little (6, 55%)
    C. Somewhat helpful (3, 27%)
    D. Very helpful (1, 9%)

17. How much do you feel the beach cleanup helped with the plastics problem (globally)?
    A. None (2, 18%)
    B. Very little (7, 64%)
    C. Somewhat helpful (2, 18%)
    D. Very helpful (0, 0%)

18. How likely are you to take action to help decrease your impact on microplastics entering our environment/help remove ocean plastics?
    A. Very unlikely (0, 0%)
    B. Somewhat unlikely (1, 9%)
    C. Neutral (0, 0%)
    D. Somewhat likely (6, 55%)
    E. Very likely (4, 36%)

19. What can you do as an individual to help with the plastics problem? (short answer)

“recycle properly and encourage others to recycle properly, reduce my footprint by purchased products that use little to none plastic”

“At a research level work to develop new technologies and approaches for plastic materials. At a personal level reduce my consumption of single use plastics and use more sustainable alternatives when possible. Additionally, find and engage with local recycling organizations to make sure I am providing collection agencies with the appropriate materials. Furthermore, call my local representatives and emphasize the importance of well informed legislation for materials sustainability as well as help inform others when appropriate.”

“use a lower level of plastic. reuse plastic items directly where possible. recycle if possible and avoid contamination of the recycle steam with non-recyclable items. participate in clean-ups (maybe closer to where I live so that I don't pollute more than I depollute in the process of getting to the cleanup site)”

“Recycle my trash”

“I can incorporate recycling into my household as well as try not to buy as many plastic items. For example, trying to use paper cups or glass bottles rather than plastic and styrofoam.”

"Reduce, reuse, recycle is of course the old adage. Here in southern MS I am finding it very hard to recycle plastics so I primarily focus on the first 2 options. In addition, as researchers we can target our studies toward helping with the plastics problem whenever possible."

"Recycle and purchase from companies who recycle"

"Be cautious of what plastics I use and try to reduce how much I use."

"While you can't control what others do, you can: opt to recycle your own waste, regularly use a reusable water bottle, opt out of using a straw at a restaurant, participate in local clean-ups, etc."

COMMUNITY CLEANUP QUESTIONNAIRE (Pre-Activity)

Number of participants: 19

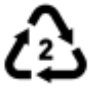

1. What do these symbols mean? (number inside chasing arrows)
   A. The product is made of recycled plastic (6, 32%)
   B. If placed into a recycling bin, the product will be recycled (1, 5%)
   C. **Identifies the type of plastic the product is made of** (4, 21%)
   D. How many times the product has been recycled (8, 42%)

2. All polymers can be recycled.
   A. True (5, 26%)
   B. **False** (14, 74%)

3. How long can plastic bottles exist in a landfill?
   A. ~10 years (0, 0%)
   B. ~50 years (4, 21%)
   C. **~500 years** (11, 58%)
   D. >1000 years (4, 21%)

4. If a plastic is advertised as being compostable, should you put it in the recycling bin?
   A. Yes (5, 26%)
   B. **No** (4, 21%)
   C. I don't know (10, 53%)

5. How much do you know about microplastics?
   A. A lot (0, 0%)
   B. Some (3, 16%)
   C. Very little (13, 68%)
   D. Nothing at all (3, 16%)

6. What best describes how you feel about microplastics in the environment?
    A. Calm (0, 0%)
    B. Somewhat calm (1, 5%)
    C. Neutral (5, 26%)
    D. Somewhat worried (7, 37%)
    E. Worried (6, 32%)

7. How much do you know about plastic recycling?
    A. A lot (1, 5%)
    B. Some (7, 37%)
    C. Very little (8, 42%)
    D. Nothing at all (3, 16%)

8. What best describes how you feel about the efficacy of recycling?
    A. Satisfactory (1, 5%)
    B. Somewhat satisfactory (5, 26%)
    C. Neutral (8, 42%)
    D. Somewhat inadequate (3, 16%)
    E. Inadequate (2, 11%)

9. What do you think some challenges of recycling are? (short answer)

    "Having to learn about it, separating it and finding out where to put it, getting the funding for it to actually happen"

    "Not knowing how to categorize items"

    "A challenge of recycling would be getting people really involved in recycling"

    "Putting bottles in recycling bin instead of trash"

    "Having people actually put plastic in recycling bins, as well as having producers and companies actually focus on recycling things, along with having cities recycle more than 1 or 2 types of material"

    "Not everyone is on board with the idea, so the problem will continue"

    "There's a lot of rules and they vary based on where you live and recycling bins are not always accessible"

    "People being too lazy to do it and put it in the recycle bin"

    "Knowing where to recycle different things"

    "The challenges are getting to people to actually do it."

    "What can/can't be recycled is unclear and the thing where they throw away a whole bunch of recyclable stuff over one piece of trash"

“Lots of items can be contaminated by one small thing and be rejected”

“Getting everyone to do it”

“No one knows how to recycle or teach people how to do it.”

“People are not putting plastics and objects that need to be recycled in the recycling bins, mass dumping of waste”

“I think a big challenge is accessibility. Even in my dorm we have recycling, but many staff members do not know where it is. I am convinced many students do not know that they can request a recycling bin.”

“placement of non-recycle in recycle bins”

“forgetting that you actually need to recycle”

“Some challenges of recycling include ensuring that it is properly recycled and not just dumped into a landfill, getting people to take initiative, and teaching what is and isn’t recyclable.”

10. What can you do as an individual to help with the plastic waste problem? (short answer)

“Help clean the environment, not use or reuse certain things, recycle”

“learn more about it so I can teach others and also be mindful of my waste”

“I could recycle”

“use paper”

“Refrain from littering and putting plastic waste in the wrong places”

“put my bottles and cans in the recycling bin”

“Try to reduce, reuse, and recycle when if you can, pick up trash on the ground as you walk by, and research recycling rules at least a little bit”

“recycle I suppose”

“recycle”

“pick up plastic that is on the ground”

“Use less plastic, repurpose plastic, and recycle plastic after use”

“recycle”

“I’ve heard of limiting plastic use, and placing trash in a plastic bottle.”

“I could sort out my garbage.”

"Put all my plastic bottles in the recycling bins"

"I try to buy reusable water bottles and do things like that to reduce plastic waste. I could also try harder to recycle more."

"use more items that can be recycled"

"recycle"

"To help with the problem of plastic waste, an individual can minimize plastic usage on all fronts and make sure to properly recycle when plastic usage is absolutely necessary so that that waste is created into a new product and not left to degrade in a landfill."

COMMUNITY CLEANUP QUESTIONNAIRE (Post-Activity)

Number of participants (total): 19

Number of participants that attended all components of the activity: 13

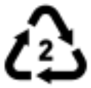

1. What do these symbols mean? (number inside chasing arrows)
    A. The product is made of recycled plastic (1, 5%) (0, 0%)
    B. If placed into a recycling bun, the product will be recycled (0, 0%) (0, 0%)
    C. **Identifies the type of plastic the product is made of** (14, 74%) (11, 85%)
    D. How many times the product has been recycled (4, 21%) (2, 15%)

2. All polymers can be recycled.
    A. True (3, 16%) (3, 23%)
    B. **False** (16, 84%) (10, 77%)

3. How long can plastic bottles exist in a landfill?
    A. ~10 years (0, 0%) (0, 0%)
    B. ~50 years (2, 11%) (0, 0%)
    C. **~500 years** (12, 63%) (10, 77%)
    D. >1000 years (5, 26%) (3, 23%)

4. If a plastic is advertised as being compostable, should you put it in the recycling bin?
    A. Yes (8, 42%) (5, 38%)
    B. **No** (8, 42%) (6, 47%)
    C. I don't know (3, 16%) (2, 15%)

5. How much do you know about microplastics?
    A. A lot (3, 16%) (2, 15%)
    B. Some (11, 58%) (10, 77%)
    C. Very little (4, 21%) (1, 8%)
    D. Nothing at all (1, 5%) (0, 0%)

6. What best describes how you feel about microplastics in the environment?
    A. Calm (0, 0%) (0, 0%)
    B. Somewhat calm (1, 5%) (0, 0%)
    C. Neutral (5, 26%) (3, 23%)
    D. Somewhat worried (7, 37%) (6, 47%)
    E. Worried (6, 32%) (4, 30%)

7. How much do you know about plastic recycling?
    A. A lot (6, 32%) (5, 38%)
    B. Some (10, 52%) (8, 62%)
    C. Very little (3, 16%) (0, 0%)
    D. Nothing at all (0, 0%) (0, 0%)

8. What best describes how you feel about the efficacy of recycling?
    A. Satisfactory (3, 16%) (3, 23%)
    B. Somewhat satisfactory (10, 52%) (8, 62%)
    C. Neutral (3, 16%) (2, 15%)
    D. Somewhat inadequate (1, 5%) (0, 0%)
    E. Inadequate (2, 11%) (0, 0%)

9. What do you think some challenges of recycling are? (short answer)

    "People actually doing it"

    "remembering to do it and not having recycling bins available"

    "The fact of people not actually putting the recycle items where they need to go."

    "remembering to actually recycle"

    "Sorting through what is actually recyclable or not."

    "Accessibility and some items are made with a recyclable and a non which makes it difficult."

    "Getting people to participate."

    "Getting people to actually care."

    "Global warming and health concerns"

    "How no one is teaching the younger generations. Why is it at my age I am finally being taught."

    "I think many people have accessibility problems."

    "Accessibility and affordability. $8/month for a recycling service? Ouch!"

    "Some challenges of recycling are: global issue, access to recycling, initiative, and remaining anti-recycling materials."

“Accessibility to various places that will take certain products -> some places that take glass or plastic bags are not widely accessible and misconceptions”

“I think that the challenge is people don’t know what can and cannot be recycled.”

“People being uneducated on it”

“willingness to participate and manufacturers aren’t going to change”

“Challenges are availability of recycling certain plastics in certain areas and the efficiency of recycling methods.”

“Knowing what places accept what.”

10. What can you do as an individual to help with the plastic waste problem? (short answer)

“recycle probably”

“make more of an effort”

“recycle more often”

“make a habit of recycling anything needing recycling”

“Clean up any trash I see on the ground.”

“recycle when I can”

“I could pick up trash when I see it”

“Do my part and recycle properly”

“sort what needs to be recycled”

“Try to recycle more.”

“I can apply to some of the programs”

“Recycle instead of throwing things away - sell or give away old clothes - keep boxes.”

“Recycle and clean up, make sure not to waste anything because it can be reused.”

“Try to recycle and encourage and educate others”

“I can start picking up trash when I see it even if its not mine”

“Not liter and recycle what I can”

“use less plastic, and do my part to recycle”

"I can understand what can and can't be recycled and can encourage others to recycle."

"Educate myself on how to recycle"

11. How much do you feel the cleanup helped with the plastic waste problem (locally)?
    A. None (1, 5%) (1, 10%)
    B. Very little (4, 24%) (2, 18%)
    C. Somewhat helpful (8, 47%) (5, 45%)
    D. Very helpful (4, 24%) (3, 27%)

12. How much do you feel the cleanup helped with the plastic waste problem (globally)?
    A. None (5, 29%) (2, 18%)
    B. Very little (6, 35%) (5, 46%)
    C. Somewhat helpful (3, 18%) (2, 18%)
    D. Very helpful (3, 18%) (2, 18%)

13. How likely are you to take action to help decrease your impact on microplastics entering our environment?
    A. Very unlikely (0, 0%) (0, 0%)
    B. Somewhat unlikely (2, 12%) (0, 0%)
    C. Neutral (4, 24%) (3, 27%)
    D. Somewhat likely (9, 52%) (6, 55%)
    E. Very likely (2, 12%) (2, 18%)

14. If the authors were to do this lecture series and cleanup again, do you have any suggestions/comments for us to improve the experience?

"No, I had a pretty good time"

"none"

"No suggestions"

"make it more engaging. the slides are a bit boring."

"No, you guys did great"

"No"

"More time spent on the cleanup would be good."

"N/A – Great project!"

"no I think y'all did great, I actually had fun."

"The lecture was extremely detailed, almost too much which made it hard to follow."

"I do not have any suggestions"

"I really liked it. I wish we were more educated on what can be recycled."

SLIDE INFORMATION

It is suggested that the following slides be used for a less informed audience:

- Pre-cleanup slides: 1-8, 10,11, 13-22, 24, 25
- Post-cleanup slides: 26, 28-34, 39

It is suggested that the following slides be used for a more informed audience:

- Pre-cleanup slides: 1, 2, 4-**12***, 13-23, 25
- Post-cleanup slides: 26, 27-39

*Slide 12 was originally used for the less informed audience, however it is instead recommended to be used with a more informed audience.

While the authors recommend specific slides for differently informed audiences, all slides are provided and can be adapted to your specific audience and needs.
